# New marketing strategies for online group-buying business from a social interaction theory perspective

**DOI:** 10.3389/fpsyg.2022.953799

**Published:** 2022-09-06

**Authors:** Lu Jiang, Yu Huang, Hong Zhu, Yingru Zou

**Affiliations:** ^1^School of Management, Nanjing University, Nanjing, China; ^2^School of Management, Jiangsu Open University, Nanjing, China; ^3^School of Business Administration, Shanxi University of Finance and Economics, Taiyuan, China; ^4^School of Management, Boston University, Boston, MA, United States

**Keywords:** online group-buying, social interaction theory, perceived trust, perceived benefits, perceived quality, purchase intention

## Abstract

Companies that use online group-buying to get new business expansion opportunities at a price advantage are failing. Therefore, there is a need to develop new marketing strategies for group-buying companies to achieve market share and consumer favor. Given that consumers are society members, we used the social interaction theory to investigate the combination of factors that stimulate consumers’ purchase intentions. Fuzzy-set Qualitative Comparative Analysis was performed to evaluate different strategy configurations of social interaction elements, perceived quality, benefits and trust to promote purchase decisions from 406 group-buying consumer questionnaires. We revealed four pathways with different configurations that can prompt consumers to make group-buying decisions: information strategy, Word-of-Mouth strategy, sense of community strategy, as well as combining Word-of-Mouth and sense of community strategy. These strategies provide viable approaches through which group-buying companies can rationally use marketing programs to promote consumers’ purchase intentions.

## Introduction

In the global business-to-consumer (B2C) industry, online group-buying (OGB) occupies an important role after birth, quick growth, rapid decline, death, and slow recovery in recent two decades (Lim, 2014b,^2015^, 2017b,^2020^; Lim and Ding, 2014). From a business perspective, OGB aims at putting online customer shopping together with a low transaction price or deep discount (Aron and Anand, 2014). For all parties, the benefits of group buying business include: consumers profit from low product costs; OGB companies benefit from expanding their sales market and acquiring new customers; and online sales platforms benefit from commissions and stable clients (Lim, 2020). Low prices is the main factor that promotes the choice of OGB by consumers, and it is also the reason for the prosperity of OGB businesses (Shiau and Luo, 2012). For example, Groupen, the earliest and biggest OGB business generated $34 million in gross billing in 2009, which increased to $5.4 billion in 2011, a compounded annual growth rate of 440% (Groupen, 2012). In 2011, the Chinese OGB market had grown by 450% (IiMedia Research, 2021).

However, low price as the original dynamics of group buying that made it successful are somewhat lost in today’s group buying and the later stages of OGB development are associated with various limitations. On the one hand, opportunities and challenges in OGB group buying may differ in some ways across different types of businesses (Lim, 2017a). For example, Lim (2017a) pointed out that businesses that are mainly physical products sellers, extending their consumer base and selling other goods on OGB websites is difficult. In contrast, for businesses that primarily provide services are more likely to generate benefits from all the identified opportunities. Therefore, it is difficult for companies to pursue discount strategies and ignore finding a suitable business model to reap the rewards of online group buying. On the other hand, excess cost-cutting in product unit price prevents OGB businesses from gaining profits, leading to their closures. Moreover, other companies cut prices to compete for the market share, which erases the advantages of OGB companies. Statistics showed that Groupen’s gross billing decreased to $4.6 and $2.6 billion in 2019 and 2020, respectively (Groupen, 2020). Therefore, OGB companies are looking for other ways to gain profits and sustained growth performance.

Studies have evaluated the business model and consumer behaviors of OGB businesses. Early studies on OGB focused on the effects of prices on consumer buying intentions, which is the main motivation for purchases, and dynamic pricing models became the main mechanisms of explanation (Kauffman et al., 2010). As a result of advances in online technologies, OGB enterprises use price discrimination strategies to attract new clients (Narasimhan, 1984). However, maintaining low-price sales approaches for extended periods may harm profits. Therefore, OGB businesses offer low-cost services to new clients while boosting pricing for the existing customers. To protect consumer interests, the government is cracking down on price discrimination induced by applications of internet technologies. Thus, OGB enterprises cannot just rely on dynamic pricing tactics to attract new clients, they should explore other marketing strategies to increase marketing sales.

Given that the price strategy fails to provide a long-term competitive advantage for OGB businesses, studies have evaluated OGB purchase intents from the consumer’s perspective, consumer involvement and consumer characters. First, some studies assessed consumers’ perceptions of the quality and ease of use of OGB websites, perceived product quality, and perceived risks to evaluate consumer satisfaction with OGB and the degree of continued purchase (Lim and Ding, 2014; Lim, 2020). Word-of-mouth (WOM) communication, a kind of interpersonal influence (Lim and Brown-Devlin, 2021), is an effective advertising method for OGB (Lim, 2015) that makes consumers perceive social trust (Suki and Suki, 2017) to seize the OGB market. Moreover, the WOM is vital as the formation of buyer aggregation is quite necessary for online group buying in the online space (Lim et al., 2022). More specifically, buyers cannot make a successful online group buying when the minimum number of buyers needed are not met. Thus, Lim et al. (2022) stated that consumers need to participate in and give prepurchase eWOM so as to persuade other buyers to join the aggregation group of online consumers to extend their opportunities of achieving a successful online grouping buying. Nowadays, some OGB businesses have helped consumers reach the minimum number of buyers as soon as possible by sending relevant opportunity vouchers directly or participating in OGB as a “stranger” (Li et al., 2022). Some studies have classified purchase psychological motivations for consumers, such as altruism, reciprocity, and reputation (Shiau and Chau, 2015); or consumer characters (e.g., variety-seeking and compulsive buying behavior) to study consumer satisfaction or purchase intentions (Lim, 2017b). Therefore, research focus has switched from OGB corporate promotion strategies to consumer-centric purchase intentions.

The impact of social forces on consumer behaviors cannot be overstated. In the dynamic pricing strategy, consumers are treated as rational persons. When confronted with similar-brand products, buyers select lower-priced options. However, consumers are social beings, and their purchasing decisions are influenced by various social interactions. During the communication process, consumers easily perceive the value and quality of the product or service. The nature and mode of brand sharing or brand information spread between customers and others, as well as social benefits of engaging with other brand users as part of their daily social life, are discussed in detail. When analyzing consumption decisions, the social environment of consumers should be considered. Previous studies focused on factors that are associated with consumer OGB purchase intentions. This study explored the factors that affect consumer’s willingness to buy and ignored the combined effects of these factors. Therefore, our research questions were: From the social interaction theory perspective, which combinations of factors inform a consumer’s willingness to buy in OGB? Do all of these variables work in tandem?

To capture consumer social interactions, the complex consumer purchase psychology, and the brand’s social value, we propose a psychological mechanism that allows consumers to share, interact, and effectively cooperate with products or information, expanding the consumer market. Moreover, for OGB companies, blindly lowering prices is not conducive for profit growth and excess marketing is associated with high marketing costs. We aimed at identifying the most essential influencing elements and approaches through which OGB companies can increase consumer intentions to buy while lowering marketing expenses. This study is divided into the following sections, Section “Literature review” describes the social interaction theory, perceived platform benefits, perceived product or service quality, and perceived trust, and the conceptual model has been followed after a literature review; Section “Methodology” describes the methodology used in this manuscript; Section “Results” addresses data analysis findings; while the discussion and conclusions are provided in Section “Discussion, implications, and recommendations.”

## Literature review

### Social interaction theory

Decades of research in psychology, sociology, economics, and other disciplines have revealed that, due to social interactions, personal decision-making is influenced by observation, perception, and expectations of others’ decision-making (Cialdini, 1993). Social interaction is a two- or more-person or between two or more social group communication or transactions in online or offline channels that is a fundamental societal component (Bagozzi et al., 2007). Scheinkman (2005) defined social interaction as “special type of externality that affects individual performance by referring to group behaviors.” Due to the influence of social interactions, the individual utility is directly dependent on the choices of others in the reference group (Brock and Durlauf, 2001), and individual behaviors may also affect the constraints, expectations, and preferences of others (Manski, 2000).

The social interaction theory highlights that an individual’s utility is influenced by others (Becker, 1974). This theory explains the social influencing factors of consumers’ purchasing decisions, which are influenced by various forms of social interactions from observational learning, such as reference group effect, queuing effect, and WOM effect (Wang and Yu, 2017). Thus, social interaction is the practice of influencing others’ buying intentions and utility without actually participating in sales. Based on reference groups, social interactions include product or service shopping information sharing (Bagozzi et al., 2007), WOM (Chen et al., 2011), and a sense of community (Lobschat et al., 2013). Reference group effects include information, utility and value expressions. The information influence of reference group relates to the information sharing between people, people and groups or between groups. The utility impact of the reference group refers to an individual’s attempt to gain benefits or avoid losses by following the wishes of others. The reference group’s value expression influence is characterized by the need to connect with others or the group, and individual hopes to be accepted by the group. The impact of social interactions on customers includes information dissemination, WOM effects, and a sense of community.

#### Social interactions with information

Consumers obtain information from other people’s behaviors, which is one of the main ways through which the social interaction effect is generated (Wang and Yu, 2017). When faced with purchasing decisions, consumers become perplexed if there is a lack of or incomplete information. Therefore, it takes a significant amount of time and money to conduct an independent analysis of each option. In this case, relying on others’ information becomes a good idea (Cai et al., 2009). For example, as an important source of information, internet advertising can provide more details of company profile, online products, or services, and thus help consumer make purchase decision easily and quickly when using OGB sites (Lim, 2015). Furthermore, Zhang and Gu (2015) reported that social interaction is a dynamic information dissemination process between and among individuals and groups. Advances in information technology have improved consumer social interaction frequency and duration (Zhang and Gu, 2015). One of the product or service shopping information sharing is sharing the product promotional information (Bagozzi et al., 2007). Due to digital social media development, consumers exchange brand information in online communities (Brodie et al., 2013). Moreover, with regards to the dimension of the conversation, the way of communication between brands and consumers has changed, thus, consumers can receive or share brand information in both online and offline channels, from the unilateral output of brand concepts and information to a social media dialogue (Nyangwe and Buhalis, 2018). One of the key strategies for initiating consumer interactions in OGB involves sharing product or service information. In addition, the exchange between consumers about brand information and OGB intentions can generate benefits between consumers and sharers (Dholakia et al., 2004). For example, by receiving the shared information, the purchaser buys the goods while sharers get discount coupons.

#### Social interactions and word-of-mouth

Word-of-mouth refers to a consumer’s activity of incorporating products or services that they are interested in into their daily communication (Duarte et al., 2018). WOM marketing frequently occurs via social interactions (Chen et al., 2011). Consumers freely express their positive or negative views and wishes in communication, including their experiences of using a certain brand product, no matter how good or bad the experience is (Brodie et al., 2013). When faced with doubt and confusion in their purchase decision-making, consumers might reach out to others and communicate for assistance in re-evaluation and decision making.

Active WOM is a type of consumer advocacy (Lobschat et al., 2013). Consumers promote the brand or share the product with positive emotions on their social media, which sends an activation signal to their friends and strangers. The brands’ active emotion and volume of positive WOM have a positive effect on brand attachment and sale performance (Proksch et al., 2015). These methods are significant in developing brand-consumer relationships, brand awareness and consumer loyalty (Duarte et al., 2018). These consumer-generated contents promote consumer engagement and affect the firm’s economic performance. Satisfaction, pride, partnerships, confidence, customer loyalty and dedication lead to positive interactions (Brodie et al., 2013). Negative WOM is counterproductive.

#### Social interaction with a sense of community

Due to social interactions, individual decisions are interdependently made, which significantly impacts on group behaviors in related groups. The sense of community refers to direct or indirect social interactions between users that creates a sense of belonging or emotional attachment (Lobschat et al., 2013). McAlexander et al. (2002) reported that buyers frequently acquire brand products with the assistance of other users, establishing a brand-centric connection. Customers improve their appreciation and sense of belonging to the brand if the firm assists consumers in building a brand community. Apart from being brand loyalists, customers drawn to these brand communities operate as brand missionaries by distributing marketing messages for their companies (Lam et al., 2010). Community members can effectively improve a person’s happiness and self-esteem, therefore, some users enjoy communicating their experiences with others (Trudeau and Shobeiri, 2016). Moreover, establishing brand communities improves consumer social interactions (Cheung et al., 2015). Online consumers gather together in brand communities that exchange social information, develop and compile group-specific meanings, negotiate group-specific identities in the society, promote the formation of social relations and create norms that serve to organize interaction and maintain desirable social climates. Then, the sense of community can enhance consumers’ OGB intention through these ways (Lim, 2014a).

### Psychological perception

The psychological state and subjective feeling of consumers’ perception of product quality or service quality have always been the research focus of consumers’ psychological perception. Zeithaml et al. (1988) stated that perceived quality means the subjective evaluation based on the sensory experience of commodity brand, quality, price and packaging, which can affect consumers’ subjective judgment on product quality, reliability, and durability. Perceived quality is an important influencing factor for consumers to make purchase decisions (Wang et al., 2021).

Moreover, Zeithaml (1988) pointed out that perceived benefits are the final results of the interaction between consumers and product providers in a certain situation, and it is an important factor in consumers’ purchase behaviors. Besides, trust is the cornerstone of a well-functioning modern society, a catalyst for market activity and an indispensable prerequisite for transactions (Pavlou, 2003). Therefore, strengthening the research on consumers’ perceived trust will help OGB enterprises better understand consumers. Thus, the psychological perception in this study is divided into three perspectives: one is the perception of the quality of products or services. The second is consumers’ perception of benefits. The third is consumers’ perceived trust in products or services.

#### Perceived product quality and e-commerce

Studies have assessed perceived products and service quality in online environments (Lee et al., 2016) or e-commerce websites and their applications (Kuan et al., 2008). Perceived quality refers to overall expectations of product quality before purchasing, which is perceived through prices (Bowbrick, 1980). Good-perceived quality goods tend to have a greater market share (Fernandes and Solimun, 2018). Oftenly, OGB attracts customer purchase intentions through low prices or deep discounts, however, it does not mean the quality is bad. Therefore, we evaluated consumer perceptions of the quality of OGB products or services and the relationship between perceived quality and purchase intentions. If e-commerce shopping malls sell the same products, the online mall’s service quality becomes a crucial factor in influencing consumer shopping decisions. For instance, the accuracy and satisfaction of customer services; the ease of searching for online products or services; and application page design aesthetics (Lee and Lin, 2005). There is a common phenomenon that product or service quality is usually asked when customers communicate. Some user-generated content entices users by evaluating that the product’s quality is superior to that of brand promotion (Chen and Roma, 2011). OGB is generated between consumers’ interactions, and good user-generated content is conducive for the development of the OGB business.

#### Perceived benefits

Perceived value plays a significant role in consumer purchasing decisions (Lim, 2020). Consumer perceived value refers to the fact that when consumers purchase products or services, they can evaluate the relative advantages of the acquisitions (Dodds and Monroe, 1985; Zeithaml, 1988). Consumers’ perceived benefits come from online purchase advantages or satisfaction of their requirements or aspirations (Lim, 2020). Zeithaml (1988) and Lim (2020) reported that perception of relative advantages obtained from an acquisition when purchasing a product or service, which is also the operational definition of this article in the OGB scenario. With regards to online shopping, Delafrooz et al. (2009) assessed perceived benefits and found that perceived advantages of customers are the degree of gain or satisfaction from online purchase that meets their needs. Lim (2020) investigated the benefits of OGB services, the higher consumers consider the benefits of online community transactions, the greater they are aware of the rights to OGB purchases. Studies have evaluated e-commerce benefits from the perspective of product or service usefulness (Racherla and Friske, 2012), safety (Liu et al., 2008), enjoyment (Kim et al., 2007), and convenience (Jiang et al., 2013).

Perceived benefits are generated via interactions between consumers. Sharing of a brand’s positive information is associated with a significant impact on customer impressions (Trudeau and Shobeiri, 2016). However, perceived benefits are personalized and vary from person to person (Al-Debei et al., 2015). The social interaction function promotes the brand or shares the product with positive emotions, supports the brand’s products, and increases the potential consumers’ ability to perceive the product’s benefits.

#### Perceived trust

**FIGURE 1:**
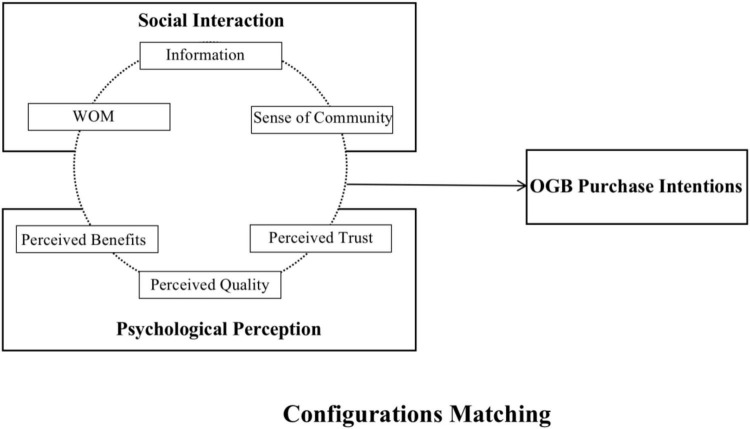
Conceptual model.

The willingness of a transaction party to have faith in and rely on other partners is referred to as trust (Koufaris and Hampton-Sosa, 2004). It is a critical component of successful customer relationship marketing (Kim and Park, 2013). Trust reduces the complexity of people’s decision-making processes and cuts on decision-making time (Siegrist and Cvetkovich, 2000). Instead of making rational decisions based on knowledge and critical thinking, people rely on trust to select trustworthy people and make decisions based on their advice (Siegrist and Cvetkovich, 2000). Compared to the traditional e-commerce, OGB adds the interpersonal trust aspect of consumers, and consumers actively seek other consumers who want to buy the product to complete the group purchase. Trust comprises four aspects: disposition to trust, institutional-based trust, trust beliefs, and trust intentions (McKnight et al., 2002). Based on literature analysis, the conceptual structure is outlined in Figure 1.

## Methodology

### Overview of fuzzy-set qualitative comparative analysis

Fuzzy-set qualitative comparative analysis (FsQCA) is a widely used research paradigm in the current business and management field (Ritala et al., 2016; Kumar et al., 2022). This is because it may not be adequate to treat the real-world context of the business phenomena as statistically symmetrical (linearly, regular frequencies) for making inferences about business environment scientifically. FsQCA considers business phenomena to be complex combinations and conceptualizes them in terms of collective relations and can be adopted to analyse the asymmetric relationships between dependent and independent variables (Woodside, 2011, 2013). Furthermore, FsQCA employs both “configuration comparison” and “set theory” in terms of methodology (Ragin, 2000). Therefore, fsQCA is involved in the complexity of the development of things, finding and identifying the causal path, leading to the same result in different situations to evaluate “multiple concurrent causality” (Rihoux and Ragin, 2008). The advantage of fsQCA is that it calculates the contribution of each configuration to precisely discover the key elements and combinations (Rihoux and Ragin, 2008). Therefore, fsQCA and the statistical approach should not be considered in the substitution perspective, but rather, in the complement perspective (Misangyi and Acharya, 2014). Given the significance of causal asymmetry test, findings from fsQCA offer a new explanation path (Woodside, 2013). In this study, fsQCA 3.0 was used to analyze which factors or combinations of factors lead consumers to OGB.

### Measures and data collection

Social interactions involve three aspects: information (4 items), word of mouth (4 items), and sense of community (3 items), as reported by Lobschat et al. (2013). One of the information items is “I can learn something new or feel fun when I communicate with this brand’s other users.” Perceived trust measures consumers’ propensity for trust group buying partners, which has 3 items (Odekerken-Schröder et al., 2003). Perceived quality refers to a consumer’s perception of the overall quality or superiority of a product and the service provided by the OGB platform, which has 4 items (Hazen et al., 2017). Finally, perceived benefits and OGB intentions come from Lim (2020), which have 3 and 4 items, respectively.

Consumers with group-buying experience were required to anonymously fill out online questionnaires. The survey was powered by www.wjx.cn. The questionnaire employed a 7-point Likert scale. To enhance the accuracy of measurement results, individuals that had not participated in group purchases were excluded. A total of 406 participants from 29 major provinces and cities in China completed the web-based survey. Among them, 182 were male (44.8%) while 224 were female (55.2%). Their demographic features are shown in Table 1.

**TABLE 1:** Demographic profile.

Variable		Count	Percentage
Gender	Male	182	44.8
	Female	224	55.2
Age	18∼25	133	32.8
	26∼30	119	29.3
	31∼40	125	30.8
	>41	29	7.1
Income	<3,000	48	11.8
	3,001∼6,000	160	39.4
	6,001∼9,000	156	38.4
	>9,001	42	10.3

The unit of income of the interviewee is CNY, 1 CNY ≈ 0.1501 USD.

### Data set and data calibration

It is important to properly adjust the calibration of fuzzy collection to the research itself, thus calibration should not be mechanical (Ragin, 2018). First, we adopted a direct approach and preferred qualitative breakpoints based on percentiles to ensure the rationality of variable allocation. This is because, potential data peaks can be skewed, therefore, even though they follow a standard distribution, data calibration must first be done, and prime values from the survey translated into 0–1 values after calibration (Brown, 2009). Three qualitative calibration anchors were included this study: the full member threshold was the sample value for 90%; full non-member threshold was the 10% point of the sample values; crossover was the sample value for 50% (Woodside, 2013). The sets, calibrations and descriptive statistics are shown in Table 2.

**TABLE 2:** Sets, calibrations and descriptive statistics.

Sets	Fuzzy set calibrations			Descriptive statistics					
	Full out	Crossover	Full in	Mean	SD	Min	Max	N Cases	Missing
Inf	2.750	5.000	6.750	4.877	1.393	1.75	7	406	0
WOM	3.333	4.333	6.333	4.805	1.215	1.667	7	406	0
SOC	2.667	4.667	6.333	4.546	1.345	1.667	7	406	0
Trust	2.333	4.000	6.000	3.999	1.285	1.333	7	406	0
PQ	3.429	5.000	6.289	4.926	1.027	1.857	7	406	0
PB	2.750	4.500	6.250	4.496	1.266	1.500	6.5	406	0
PI	2.250	3.750	5.500	4.877	1.393	1.750	7	406	0

Inf, Information; SOC, Sense of Community; PQ, Perceived Quality; PB, Perceived Benefits; PI, Purchase Intention.

## Results

### Statistics approach

The reliability and validity analysis are performed before performing the regression. Confirmatory factor analysis (CFA) was used to ensure the quality of construct measurement. For assessing the reliability of research measurement, the value of Cronbach’s Alpha, average-variance-extracted (AVE), and composite reliability (C.R.) were calculated. As shown in Table A1 in Appendix A, the Cronbach’s Alpha of all constructs exceeded the recommended 0.7 level (Henseler et al., 2016; Henseler, 2017), 2016); the composite reliability (C.R.) was slightly higher than the Alpha coefficient from 0.878 to 0.940, with all values above 0.6; the values for average variance extracted (AVE) were between 0.705 and 0.797, which were higher than the suggested 0.5 (Hair et al., 2013). Consequently, results show satisfying reliability. As depicted in Table A1 in Appendix A, the square root (in bold) of the AVE value for each construct is higher than their Pearson correlations (Fornell and Larcker, 1981). These results indicate that each construct is empirically unique and reveals sufficient discriminant validity (Sarstedt et al., 2022). After ensuring reliability and validity, regression analysis was performed, and the results are shown in Table B1 in Appendix B.

### Analysis of necessary conditions

Before the sufficiency analysis, necessity conditions analysis was performed to determine whether there are necessary conditions to motivate consumers to use OGB services. Outcomes from the necessary conditions test for purchase intention are shown in Table 3. There was no consistency higher than 0.9, implying that all elements are not necessary conditions for purchase intentions. Therefore, the impact of conditional designs on consumer purchasing intentions should be investigated.

**TABLE 3:** Analysis of necessary conditions.

Outcome variable: Purchase Intention Conditions tested:		
**Variable**	**Consistency**	**Coverage**
Information	0.655	0.637
∼Information	0.574	0.588
WOM	0.680	0.646
∼WOM	0.542	0.570
Sense of community	0.663	0.654
∼Sense of community	0.575	0.581
Perceived trust	0.667	0.705
∼Perceived trust	0.588	0.556
Perceived quality	0.764	0.751
∼Perceived quality	0.493	0.500
Perceived benefits	0.736	0.722
∼Perceived benefits	0.502	0.510

### Analysis of sufficient conditions

Building the configurations for achieving high levels of outcome conditions are shown in Table 4. It is shown that there are 4 solutions for encouraging consumers toward OGB purchases. A consistency threshold should not be below 0.75 (proportional reduction in inconsistency ≥ 0.75), while the raw consistency benchmark should be greater than 0.8 (Greckhamer et al., 2018). Findings from fsQCA 3.0 analysis show that three solutions (i.e., Standard, Parsimonious and Complex) output the configurations (Fiss, 2011). We report on intermediate solutions because the important benefit of intermediate solutions is that they do not allow the removal of necessary conditions (Rihoux and Ragin, 2008). Table 5 shows the configurations for the absence of the outcome conditions (fsQCA). The results display that although there are three paths leading to absence, it is meaningless.

**TABLE 4:** Configurations for achieving high levels of the outcome conditions (fsQCA).

Causal configuration	S1	S2	S3	S4
Information	🌑			⊗
WOM		🌑		🌑
Sense of community			🌑	🌑
Perceived trust	🌑	∙	∙	
Perceived quality	🌑	∙	∙	🌑
Perceived benefits	🌑	∙	∙	🌑
Raw coverage	0.392	0.387	0.390	0.236
Unique coverage	0.021	0.019	0.013	0.032
Consistency	0.879	0.879	0.887	0.922
Solution coverage: 0.491				
Solution consistency: 0.874				

🌑, presence core conditions; ⊗, absence core conditions; ∙, present contributing conditions; ⊗, absence contributing conditions; blank, do not care.

**TABLE 5:** Configurations for the Absence of the outcome conditions (fsQCA).

Causal configuration	AS1	AS2	AS3
Information	⊗		⊗
WOM	⊗	⊗	
Sense of community		⊗	⊗
Perceived trust	⊗	⊗	⊗
Perceived quality	⊗	⊗	⊗
Perceived benefits	⊗	⊗	⊗
Raw coverage	0.319	0.320	0.325
Unique coverage	0.032	0.033	0.038
Consistency	0.898	0.906	0.906
Solution coverage: 0.389			
Solution consistency:0.904			

🌑, presence core conditions; ⊗, absence core conditions; ∙, present contributing conditions; ⊗, absence contributing conditions; blank, do not care.

### Configurations for online group-buying purchase intentions

We found that the total consistency rate that promotes consumers to have OGB purchase intentions greater than the agreed threshold of 0.800 (solution consistency is 0.874) and solution coverage is 0.491. Four configurations promote consumers to have OGB purchase intentions. Among the 4 solutions, perceived benefits and perceived quality are significant. First, the information strategy, which comprises information, trust, perceived benefits, and perceived quality, is solution 1 (S1) for improving the purchase intent. The basic function of social interactions is information transmission. The information strategy should encourage consumers to share OGB information. Consumers and sharers can benefit from each other’s brand information and collective buying intents, resulting in mutually beneficial and win-win outcomes. The consistency of S1 (0.879) approaches agreed with the threshold of 0.800, as shown by its raw coverage (0.392). Information exchange is useful when information is scarce. However, consumers have more channels for obtaining brand information, and pure information exchange has limited stimulation of consumers’ willingness to shop in the context of the proliferation of Internet information. In social interactions, consumers’ WOM communication is mixed. Social interaction is one of the most powerful forms of advertising as consumers trust their friends or other consumers over traditional advertising. Solution 2 (S2) is the WOM strategy, which shows the importance of consumers’ positive WOM and user-generated content that send an activation purchase signal to their friends and strangers. The S2 configuration includes WOM, trust, perceived benefits, and perceived quality. Raw coverage and consistency of S2 were 0.387 and 0.879, respectively.

Apart from perceived quality and perceived benefits, solution 3 (S3) is the sense of community approach, which emphasizes on the significance of the sense of community, trust, perceived benefits and perceived quality. With regards to psychological needs, the basic assumption of social interaction is that consumers are societal members who require a sense of belonging in the community. A well-run brand community can meet this psychological need. The significance of customer interactions in brand community is emphasized in the community strategy perspective. Consumers can immediately evaluate quality, perceived benefits, and create trust relationships by frequently communicating in online or offline communities, which facilitates purchases. Respectively, raw coverage and consistency are 0.390 and 0.887. It is more difficult for a brand to gain consumer trust in a short period, and consumers are prone to forgetting promotional information. Social interaction strategies provide brands with a flexible solution to stimulate consumer purchase intentions while not explicitly pursuing consumer trust and without deliberately promoting brand promotion information. Solution 4 (S4) offers WOM and the sense of community strategy for consumer purchase intentions, including WOM, communication with community members, perceived quality and perceived benefits. Although there is no direct product information, when consumers advocate products to each other in communication and make people aware of the perceived benefits and quality, purchasing intention can be stimulated.

### Robustness checks

Robustness checks in fsQCA research are necessary. In cases of a lack of attention to robustness testing, findings from fsQCA analyses are frequently questioned (Schneider and Wagemann, 2012). For robustness testing, we employed a set theory-specific method (changing consistency and frequency thresholds). Schneider and Wagemann (2012) reported that throughout the reducing process, the selected raw consistency threshold can impact the number of rows (configuration number) of the truth table, influencing the final outcomes. To increase the consistency threshold, the number of truth tables included in the minimization analysis should be lowered, as are the number of cases. Therefore, optimization of the simpler configuration is challenging. Eventually, the new configuration will be a subset of the adjusted configuration. First, the frequency cutoff is increased from 1 to 2 in the study. The conclusion is unchanged, and the robustness test is successful. Second, Based on the analytical method from Ordanini et al. (2014), the raw consistency threshold should be raised by 0.05. In this study, instead of the 0.8 raw consistency threshold, 0.85 is used, and the study is repeated with a stricter threshold. The conclusion remains intact, demonstrating that our findings are robust.

## Discussion, implications, and recommendations

### Theoretical contributions and implications

For the first time, using a configuration model, we investigated the contextual configuration path of social interactions and product perception to consumers’ OGB purchase intentions, which differs from previous single and fragmented research findings. Based on the social interaction theory, factors influencing consumer purchase intentions are dynamically changing. The success of an OGB business requires open and interactive consumer interactions. Therefore, the social interaction theory can be applied to understand consumer purchase intentions for OGB. Studies should be grounded in the real world to serve real brands or companies from a holistic perspective, rather than simply proving theoretical assumptions (Harley, 2019). Based on the premise that consumers are societal members, this study differs in that it considers company marketing strategies and consumer perceptions as multiple reasons for stimulating consumer purchase intentions, and it employs the social interaction theory to investigate the combination of factors that stimulate consumer purchase intentions. It also proves the importance of social interactions for brand marketing strategies from complexity and causal asymmetry perspectives. Our findings provide novel ideas for future studies on consumer purchase intents.

Moreover, we demonstrate that implementing all social interaction tactics in tandem is a waste of resources and that focusing on one or some elements for brand operation is an effective strategy for attracting consumers to shop. Based on the causal complexity perspective, we assessed the influence of the social interactions concept toward OGB, and the effects of consumers’ perceived trust, benefits and quality on OGB intention. We explored social interactions about information sharing, WOM, and sense of community (Lobschat et al., 2013), and highlight consumer psychological perception about perceived trust, quality and benefits (Lim, 2020). Based on the fsQCA approach, we found that there are four solutions for promoting OGB purchase intentions, which lays forth a strategy for increasing consumer purchasing intent in various situations. Perceived quality and perceived value are important in the four configuration paths for group buying business, consistent with previous research. The difference is that this study discusses whether consumers perceive trust or not for group buying research. The first three solutions address consumer perceptions of trust as well as consumer purchase intentions. The fourth solution path allows group buying companies to prompt consumers to make purchases without sending specific sales information or gaining consumer perception and trust.

Third, in terms of research method innovation, we used fsQCA that is tailored to a complex environment. There is a need to develop new research methods to adapt to the changing market environment. Most current studies on OGB use linear regression analyses to assess the correlation and net effects of simple factors on consumer purchase intentions, which makes it difficult to analyze how to stimulate consumer purchase intentions with several factors. For example, according to our regression results, WOM and the sense of community have a non-significant effect on purchase intention. Moreover, the effect of information on purchase intention is negative. These are because regression analysis studies the net effect between variables. When the regression paths are found to be non-significant, scholars tend to overlook the synergistic effects of variables, but fsQCA helps to fill this gap. From the regression results, perceived benefits and perceived quality are significant elements; to the results obtained in our study, all of the configurations S1, S2, S3, and S4 include perceived benefits and perceived quality. Furthermore, information has a negative effect on purchase intention, which is consistent with S4 (S4 = ∼Inf*WOM*SOC*PQ*PB). Although neither WOM nor sense of community has a significant effect on purchase intention according to the regression results, when combined with other conditions, WOM and sense of community can appear as core conditions., as in S2 (S2 = WOM*Trust*PQ*PB) and S3 (S3 = SOC*Trust*PQ*PB). Researchers may miss these valuable results if they only concern the regression analysis.

### Implications for managers

The ultimate goal of businesses is to reduce costs while increasing revenue. Companies that use online group-buying to get new business expansion opportunities at a price advantage are failing. This necessitates the development of new marketing strategies by group-buying companies to improve the market share and consumer favor. However, blindingly putting all marketing elements into the market to increase market share is a waste of resources. Moreover, it is unwise to reduce a certain part of marketing investment at will. Our findings provide specific solutions for brand social interactions in different research scenarios. The key finding of this study is that OGB enterprises that require consumer cooperation to accomplish OGB activities can still employ various perspectives of social interactions as a marketing strategy to boost consumer willingness to buy: by focusing solely on information-sharing strategy (S1), or WOM strategy (S2), or the sense of community strategy (S3), or combining sense of community and WOM strategy (S4).

Using a social interactions strategy that focuses solely on one component, as long as customers feel the trust, good quality perception, and perceived benefits, they can ignite potential consumers’ desire to buy. This feature is primarily reflected in the first three solutions. The information-sharing strategy (S1) highlights the role of product information sharing. Sharing the brand’s related information online is a key part of OGB because merchants try their best to keep their sales information from being overwhelmed, and when surrounded by massive online information, customers prefer to quickly find useful information. Based on online technologies, sharing, and disseminating information is easier than before. It is very important to share information since all advertisements or product information would be shown when customers click the link. Highlighting quality and value in information and winning consumer trust is an important combination of factors that motivate consumers to buy.

The WOM strategy (S2) emphasizes the role of advocacy in promoting consumer perceptions of trust, quality, and value. WOM marketing can be fostered through various online celebrity endorsements, as well as ordinary people’s efforts to encourage consumer-to-consumer interactions and purchase intents. In social interactions, fans can unwittingly transmit brand trust and value to potential consumers. The role of fans is also reflected in the community strategy (S3) perspective. For example, enthusiastic fans are the most engaged consumers. They give other consumers a sense of belonging and community, which makes them feel like the same sort of individuals. The sense of community strategy emphasizes the role of users’ sense of belonging.

Finally, the research provides a marketing strategy (S4) for social interactions if gaining consumer trust proves challenging. In this scenario, product information is not directly advertised, but the importance of the sense of community and active WOM is stressed to persuade consumers to perceive the brand’s quality and value, and ultimately to urge consumers to purchase. Perceived value and perceived quality are components in all four solutions, implying that when companies use the brand’s social interaction for advertising or promotion in OGB, enterprises should highlight brand quality while promoting perceived benefits. They all encourage a rise in customer purchase intentions. To reduce marketing costs and increase revenue, businesses should select appropriate solutions based on their market environment and target customer positioning. Table 6 shows the clarity of the implications of the findings to both theory and practice.

**TABLE 6:** Key implications of the findings.

Strategy	Configuration	When to use	How to implement
S1	Inf*Trust*PQ*PB	No matter whether WOM is active or not, sense of community is high or low, S1 strategy can be implemented	OGB businesses should encourage consumers to share OGB information. Consumers and sharers can benefit from others’ information and collective buying intents, resulting in mutually beneficial outcomes
S2	WOM*Trust*PQ*PB	Whether consumers have access to information or not, sense of community is high or low, S2 strategy can be implemented	OGB businesses need to realize the importance of advocacy in promoting consumer perceptions of trust, quality, and value. Companies can invite online celebrity to endorse, encourage ordinary people to interact, so as to promote consumers’ purchase intention
S3	SOC*Trust* PQ*PB	Whether consumers have access to information or not, WOM is positive or not, S3 strategy can be used	Consumers are societal members. They can evaluate quality, perceived benefits, and create trust relationships by frequently communicating in online or offline communities, which facilitates purchases. Thus, OGB businesses should operate and maintain its brand community attentively
S4	∼Inf*WOM* SOC*PQ*PB	When consumers are not able to get direct product information, S4 strategy can be used	Consumers can advocate products to each other in communication and make people aware of the perceived benefits and quality, then purchasing intention can be stimulated. Therefore, OGB businesses should focus on building consumers’ high sense of community and active WOM

Inf, information; SOC, sense of community; PQ, perceived quality; PB, perceived benefits; PI, purchase intention.

## Conclusions, limitations, and future research directions

Due to escalating competitive pressures and rising marketing costs, OGB enterprises are experiencing bleak growth prospects. It is unreasonable to use marketing tactics to support OGB business growth without thinking about it. Based on knowledge of the features of OGB firms, adoption of the right marketing approaches can boost consumer purchasing intentions. From the social interaction perspective, we evaluated the effectiveness of OGB corporate marketing strategies based on the causal complexity logic. A data set of 406 group buying consumers were analyzed via fsQCA. We focused on the impact of social interactions as well as consumer perceptions of trust, quality, and value, on purchase intent. Various conditions resulted in formation of four final pathways to encourage customers to increase their purchasing intentions. Thus, OGB businesses can succeed by leveraging their unique business characteristics and adjusting marketing strategies to mitigate the losses associated with blind marketing investments. Based on brand community’s consumer characteristics, OGB companies should select which marketing approaches to apply and dynamically adapt the marketing strategy as the situation of the brand community’s consumers evolves.

This study also has s several limitations which presents new directions for future research. Firstly, the research results are based on cross-sectional data. In the future, methods such as situational experiments or longitudinal studies can be designed to analyze the causal relationship between variables. Secondly, this study is limited in terms of its sampled population (only in one country), which limits generalizability. Continued study could replicate this research in other countries and with other populations to investigate whether the proposed relationships still exist and to identify any possible cultural differences that may affect the impact of social interaction and psychological perception on consumers in OGB. Such research would provide OGB with more in-depth insights and increase the generalizability of results. Third, this study furnishes underpinnings for OGB only from the perspective of social interaction and psychological perception. Future research should consider other perspectives, such as artificial intelligence, machine learning in OGB (Lim, 2022).

## Data availability statement

The raw data supporting the conclusions of this article will be made available by the authors, without undue reservation.

## Author contributions

LJ and HZ conceived the presented idea. LJ designed the conceptual model and wrote the original draft of this manuscript. YH and YZ contributed to data processing and performed the analysis. HZ was involved in all decision-making process related to the manuscript. All authors contributed to the article and approved the submitted version.

## Appendix

**Appendix A:** Results of validity, reliability of measures, and correlation matrix.

Construct	Alpha	C.R.	AVE	Inf	PB	PQ	PI	SoC	Trust	WOM
Inf	0.916	0.940	0.797	0.893						
PB	0.896	0.928	0.763	0.237	**0.874**					
PQ	0.888	0.922	0.748	0.233	0.428	**0.865**				
PI	0.891	0.925	0.754	0.112	0.455	0.458	**0.868**			
SOC	0.856	0.910	0.772	0.407	0.149	0.135	0.158	**0.878**		
Trust	0.861	0.915	0.782	0.173	0.336	0.395	0.356	0.210	**0.885**	
WOM	0.793	0.878	0.705	0.477	0.223	0.179	0.196	0.461	0.194	**0.840**

Inf, information; SOC, sense of community; PQ, perceived quality; PB, perceived benefits; PI, purchase intention. The square root of the AVE is in bold.

**Appendix B:** Regression results.

Paths	β	STD	T statistics	*P* values
Information → Purchase intention	−0.106	0.046	2.323	0.020
Perceived Benefits → Purchase intention	0.286	0.046	6.198	0.000
Perceived Quality → Purchase intention	0.284	0.051	5.589	0.000
Sense of Community → Purchase intention	0.054	0.045	1.190	0.234
Trust → Purchase intention	0.138	0.045	3.060	0.002
WOM → Purchase intention	0.080	0.050	1.600	0.110
